# Longitudinal Comparison of Bacterial Diversity and Antibiotic Resistance Genes in New York City Sewage

**DOI:** 10.1128/mSystems.00327-19

**Published:** 2019-08-06

**Authors:** Susan M. Joseph, Thomas Battaglia, Julia M. Maritz, Jane M. Carlton, Martin J. Blaser

**Affiliations:** a New York University School of Medicine, New York, New York, USA; b Center for Genomics and Systems Biology, Department of Biology, New York University, New York, New York, USA; c Center for Advanced Biotechnology and Medicine, Rutgers University, Piscataway, New Jersey, USA; University of Illinois at Chicago

**Keywords:** antibiotic resistance, microbiome, New York City, sewage

## Abstract

Bacterial resistance to antibiotics is a pressing health issue around the world, not only in health care settings but also in the community and environment, particularly in crowded urban populations. The aim of our work was to characterize the microbial populations in sewage and the spread of antibiotic resistance within New York City (NYC). Here, we investigated the structure of the microbiome and the prevalence of antibiotic resistance genes in raw sewage samples collected from the fourteen NYC Department of Environmental Protection wastewater treatment plants, distributed across the five NYC boroughs. Sewage, a direct output of anthropogenic activity and a reservoir of microbes, provides an ecological niche to examine the spread of antibiotic resistance. Taxonomic diversity analysis revealed a largely similar and stable bacterial population structure across all the samples, which was found to be similar over three time points in an annual cycle, as well as in the five NYC boroughs. All samples were positive for the presence of the seven antibiotic resistance genes tested, based on real-time quantitative PCR assays, with higher levels observed for tetracycline resistance genes at all time points. For five of the seven genes, abundances were significantly higher in May than in February and August. This study provides characteristics of the NYC sewage resistome in the context of the overall bacterial populations.

**IMPORTANCE** Urban sewage or wastewater is a diverse source of bacterial growth, as well as a hot spot for the development of environmental antibiotic resistance, which can in turn influence the health of the residents of the city. As part of a larger study to characterize the urban New York City microbial metagenome, we collected raw sewage samples representing three seasonal time points spanning the five boroughs of NYC and went on to characterize the microbiome and the presence of a range of antibiotic resistance genes. Through this study, we have established a baseline microbial population and antibiotic resistance abundance in NYC sewage which can prove to be very useful in studying the load of antibiotic usage, as well as for developing effective measures in antibiotic stewardship.

## Abstract (precis)

Urban sewage or wastewater is a diverse source of bacterial growth, as well as a hot spot for the development of environmental antibiotic resistance, which can in turn influence the health of the residents of the city. As part of a larger study to characterize the urban New York City microbial metagenome, we collected raw sewage samples representing three seasonal time points spanning the five boroughs of NYC and went on to characterize the microbiome and the presence of a range of antibiotic resistance genes. Through this study, we have established a baseline microbial population and antibiotic resistance abundance in NYC sewage which can prove to be very useful in studying the load of antibiotic usage, as well as for developing effective measures in antibiotic stewardship.

## INTRODUCTION

Antibiotic resistance (AR) in bacteria is the ability of a microorganism to survive in the presence of the inhibitory activity of antibiotics. Certain bacteria possess naturally occurring or intrinsic resistance, such as vancomycin resistance in strains of Escherichia coli or the presence of β-lactamases in *Streptomycetes* (1). Alternatively, bacterial resistance to antibiotics may be acquired either via mutations that result in resistant survivors in a bacterial population (2) or via horizontal gene transfer through mechanisms including transformation or transfer of mobile elements such as plasmids, transposons (e.g., the *mecA* gene in methicillin-resistant Staphylococcus aureus [MRSA]) and bacteriophages (e.g., resistance transfer in strains of S. aureus) (3). This ability to horizontally acquire resistance or resistance genes further magnifies the threats presented by AR.

Antibiotic resistance continues to grow and is now becoming a major global health crisis (4–6). Reported antibiotic resistance has led to excess mortality and failed treatment modes in hospitals, contributing to increased health care costs (7). These rising AR levels are fueled by the lack of rigid monitoring and control of the use of antibiotics in humans (8, 9) and in domesticated animals (10–12). Further exacerbating this global problem is that AR is not restricted to clinical settings but also occurs in both domestic and natural environments (13–16).

Raw sewage or wastewater is the product of many human activities in domestic, clinical, and industrial settings. Sewage represents a conglomerate of numerous microbes, including bacterial families found in human microbiota such as *Bacteroidaceae*, *Ruminococcaceae*, and *Lachnospiraceae* (17). Many of these bacteria enter the sewage environment as carriers of AR genes and exhibit AR phenotypes. Exposure to residual antibiotics in sewage wastes can further select for resistance traits, which can then be horizontally amplified within bacterial populations. As such, untreated sewage can increase the spread of AR (16) due to the presence of both residual antibiotics and AR genes, which also can survive wastewater treatment (18). As a consequence, AR genes are now considered to be “environmental contaminants” (19).

Citywide environmental microbiome studies can serve to monitor the health of the residents of the city. New York City (NYC), the largest U.S. city, with >8.6 million inhabitants, is comprised of five boroughs: Manhattan, Brooklyn, Queens, the Bronx, and Staten Island, each with ethnically diverse populations (20). The city is home to the country’s largest health care base with numerous busy hospitals. NYC’s enormous waste disposal needs are served by an extensive sewer system, including >7,000 miles of pipes leading to 14 wastewater treatment plants distributed across the five boroughs. The majority of these sewers are combined, carrying a mix of industrial and domestic wastewater, along with storm and rain water runoffs (21).

**FIG 1:**
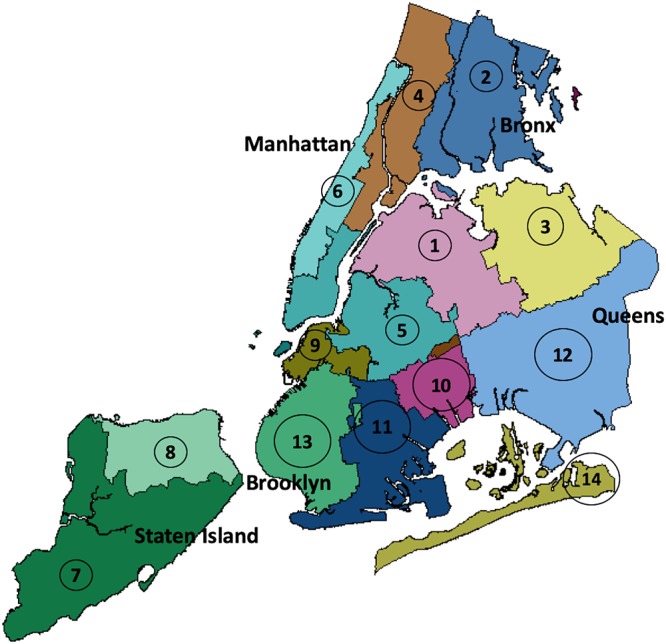
Geographical distributions of NYC WWTPs. This New York City map indicates the drainage areas for the 14 WWTPs across the five NYC boroughs from which samples were obtained for this study. The map was plotted with the Maptools package in R using GIS data obtained from Open Sewer Atlas NYC (http://openseweratlas.tumblr.com/data).

In this study, we collected 51 raw sewage samples from the 14 NYC wastewater treatment plants (WWTPs), which included serial specimens taken over a 6-month period from February to August 2015 (Fig. 1). Based on these samples, we conducted a culture-independent longitudinal investigation of the bacterial microbiome in NYC sewage and assessed the presence of clinically important AR genes. The two key questions that we sought to address were: (i) does the microbial population in NYC sewage vary between the boroughs over time; and (ii), can NYC sewage be used to monitor the spread of environmental antibiotic resistance in the city?

## RESULTS

### 16S rRNA sequence analysis of NYC sewage samples.

The two MiSeq 16S rRNA sequencing runs of the raw sewage DNA samples yielded 39,010,014 (4,083 Mb) and 53,685,483 (5,619 Mb) sequences, with 92.1 and 85.5% showing Q30 values, respectively. After sequence assembly and quality filtering using QIIME, 15,984,186 paired-end reads remained for OTU picking. The 102 sequenced samples were represented by 384 unique OTUs from the generated paired-end reads. The number of reads in each sample ranged from a minimum of 58,892 to a maximum of 222,182, with a mean of 134,364 ± 31,164 reads/sample. All further diversity analyses were performed on operational taxonomic unit (OTU) tables rarefied to 58,892 reads.

**FIG 2:**
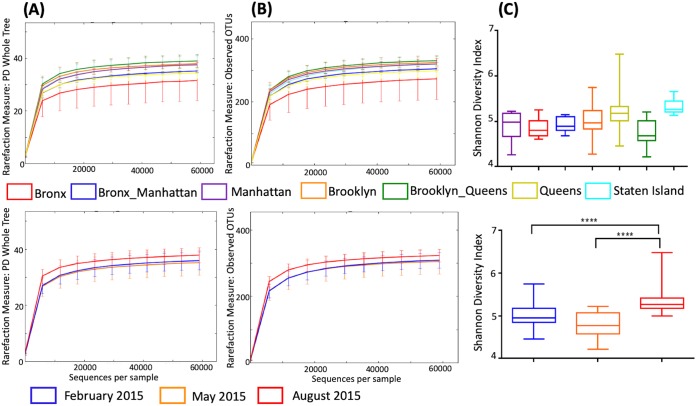
Alpha diversity in 102 sequenced sewage samples. Rarefaction was based on (A) phylogenetic diversity. (B) Observed number of OTUs. The *x* axes represent the numbers of sequences used in the rarefaction analysis. (C) Box-and-whisker plots depicting the species-level richness and diversity of OTUs based on the Shannon diversity index in the sewage samples. Within the boxes, the central line represents the median values, and the upper and lower boundaries indicate the 75th and 25th percentiles, respectively. The whiskers show the maximum and minimum values. Top panels show results based on location (NYC borough); bottom panels show results based on sampling season. Statistical significance was tested by one-way analysis of variance using the Kruskal-Wallis test, followed by multiple comparisons using Dunn’s test (****, *P* < 0.0001).

Alpha diversity analysis showed that this sequence depth provides an even sampling distribution across the population, based on both the phylogenetic diversity, as well as the observed number of OTUs (Fig. 2A and B). The Shannon diversity index had a mean value of 5.0 across the total population. Compared between sampling time points, the index was found to be significantly higher (*P* < 0.001) during August compared to February and May (Fig. 2C).

### Beta diversity analysis and batch effects.

**FIG 3:**
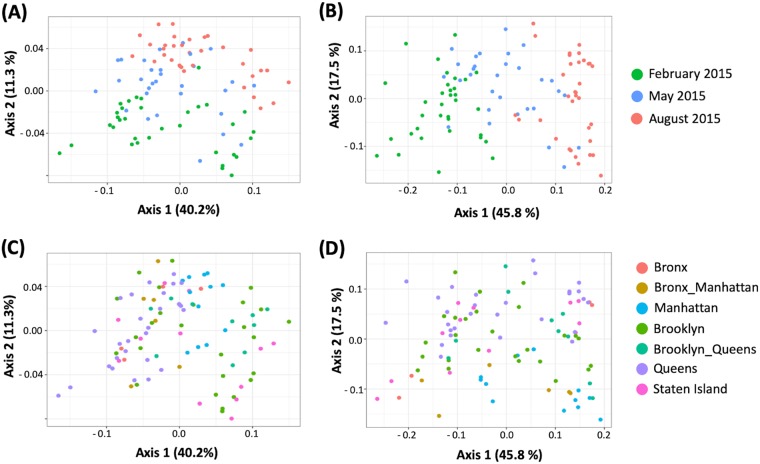
Beta diversity in sewage samples. PCoA results represent unweighted (A and C) and weighted (B and D) UniFrac distances by NYC major drainage area (lower panels) and sampling season (upper panels). Statistical significance was tested by the Adonis test using the Vegan package in R. Each of the data sets indicated distinctions between the tested groups at *P* < 0.001.

Both weighted and unweighted UniFrac distances were used to determine the beta diversity within the sample populations. Initial analyses using the OTUs generated from the open reference data set revealed a strong clustering pattern based on the two Illumina MiSeq runs, especially in the unweighted analyses (see Fig. S1 in the supplemental material). Since the technical replicates from the May samples had been split between two sequencing runs (Table S1), the OTUs for this time point were studied in detail. The novel OTUs (94%) identified as part of the *de novo* clustering algorithm during the open reference OTU picking were found to be the major factor differentiating between the replicates. When the beta diversity of only the May samples was then replotted after the exclusion of these OTUs, the clustering pattern previously observed in the unweighted UniFrac analysis was found to diminish (Fig. S2A and C). Hence, the imbalance in the *de novo* OTU sequences between the runs could explain this artifact. Repeating the diversity analyses for the entire sample set using the OTUs generated from the closed-reference database showed elimination of this technical artifact. As such, to maintain analytical uniformity, all further analyses for this study were performed using the closed reference OTU database, despite the potential loss of unique bacterial diversities. The principal coordinate analysis (PCoA) plots that were generated using the unweighted UniFrac distances showed the 102 samples to be very tightly clustered, with no significant separation due to the sequencing run or sampling season, except for a single sample (ID S050.1) from the Bronx (Fig. S3A and C). When this outlier sample was excluded from the analysis, PCoA plots generated using both unweighted and weighted UniFrac distances showed a more distributed population (Fig. 3). By PERMANOVA analysis, 22.4 and 12.5% of the variation in the population could be accounted for by sampling month (Fig. 3A) and borough of origin, respectively (Fig. 3C); these relationships were significant (*P* < 0.01) using the Adonis test for variance. Only 0.5% (*P* = 0.57) of the population structure was explained by sequencing run differences, compared to 8.4% in the analysis when the open-reference OTU picking algorithm was used (*P* < 0.001).

10.1128/mSystems.00327-19.1 FIG S1 Beta diversity in the 102 sewage samples presented using the open-reference OTU picking algorithm. PCoAs show unweighted (A and C) and weighted (B and D) UniFrac distances based on sequencing run (upper panels) and sampling month (lower panel). Statistical significance was tested with the Adonis test using the vegan package in R. Download FIG S1, TIF file, 1.0 MB. Copyright © 2019 Joseph et al. 2019 Joseph et al. This content is distributed under the terms of the Creative Commons Attribution 4.0 International license.

10.1128/mSystems.00327-19.5 TABLE S1 Details of the sample IDs and sampling and sequencing information for the sewage samples included in this study. Download Table S1, XLSX file, 0.01 MB. Copyright © 2019 Joseph et al. 2019 Joseph et al. This content is distributed under the terms of the Creative Commons Attribution 4.0 International license.

10.1128/mSystems.00327-19.2 FIG S2 Beta diversity in the technical replicates of the sewage samples. PCoAs representing unweighted (A and C) and weighted (B and D) UniFrac distances in the May replicate samples using the open-reference OTU picking algorithm (upper panels) and after exclusion of the OTUs differing between the replicate groups (lower panels). Statistical significance was examined with the Adonis test using the vegan package in R. Download FIG S2, TIF file, 1.0 MB. Copyright © 2019 Joseph et al. 2019 Joseph et al. This content is distributed under the terms of the Creative Commons Attribution 4.0 International license.

10.1128/mSystems.00327-19.3 FIG S3 Beta diversity in the 102 sewage samples using a closed-reference OTU picking algorithm. PCoA plots showing unweighted (A and C) and weighted (B and D) UniFrac distances, respectively, based on sequencing runs (upper panels) and sampling month (lower panels). Statistical significance was tested with the Adonis test using the vegan package in R. Download FIG S3, TIF file, 0.9 MB. Copyright © 2019 Joseph et al. 2019 Joseph et al. This content is distributed under the terms of the Creative Commons Attribution 4.0 International license.

### Bacterial taxonomic diversity in the NYC sewage.

**FIG 4:**
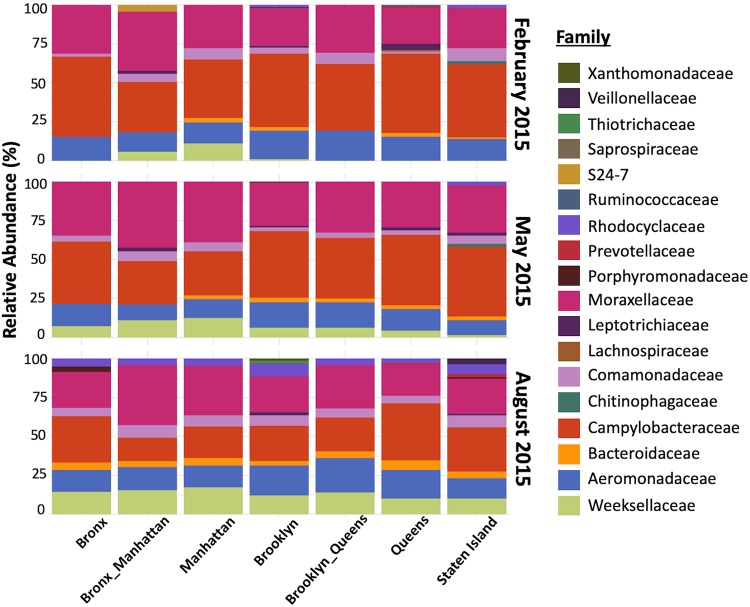
Relative taxonomic abundances of the assignments in 102 sewage samples. Samples are grouped by family according to NYC borough and sampling season. Taxonomy was assigned to the identified OTUs based on Greengenes database v13.8. Only families represented by >3% abundance are listed.

Taxonomic diversity analysis revealed a largely similar and stable population structure across all the samples and across multiple time points, as well as across the NYC boroughs, with minor variation (Fig. 4). At the phylum level, *Proteobacteria* were found to be dominant (76.4%), followed by *Bacteroidetes* (14.1%), along with smaller proportions of *Fusobacteria* and *Firmicutes;* representatives of all four phyla being present in all samples. Phyla detected in some of the samples at low abundances included *Acidobacteria*, *Actinobacteria*, *Chlorobi*, *Spirochaetes*, *Verrucomicrobia*, and WWEI (an OTU previously identified in an anaerobic sludge digester [22]) .At the family level, the OTUs were predominantly assigned to members of the families *Campylobacteraceae* (30.6%), *Moraxellaceae* (22.9%), and *Aeromonadaceae* (12.7%), all three of which were present in all boroughs at all three time points (Fig. 4). The family *Weekselaceae*, present at a relative abundance of 6.2% in the total population, was found in all the boroughs in May and August but only in 3 of 7 in February. Of the 73 families identified which comprised the 384 OTUs (indicated in Table S3), 23 were present in all 102 samples, representing the core microbiome, i.e., the organisms present in all sampled locations at all three time points, although most were present at very low abundances.

**FIG 5:**
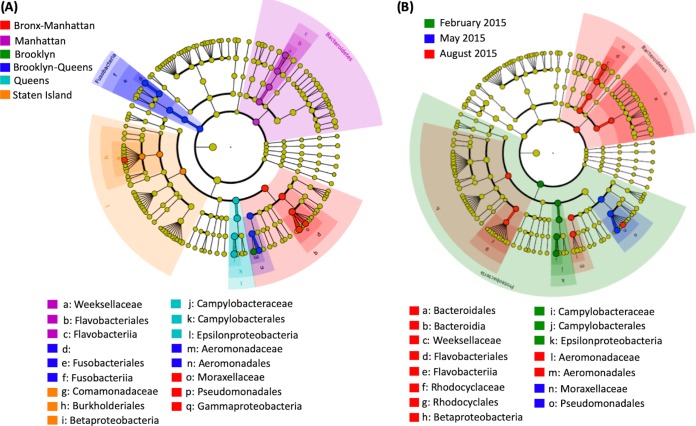
Differentially abundant OTUs identified in 102 sewage samples. LEfSe analyses were performed based on NYC borough (A) and sampling time point (B). Significantly abundant OTUs were determined based on an alpha value of <0.05 and a logarithmic LDA score (effect size) of >4.0.

The OTUs with significantly higher relative abundances in each NYC borough, as well as each sampling time point, were calculated based on a linear discriminant analysis Effect Size algorithm (LEfSe; Fig. 5). Compared by borough, Manhattan showed a significantly higher abundance of *Bacteroidetes* members as biomarker taxa, while the other boroughs had biomarker taxa belonging to the phyla *Fusobacteria* and *Proteobacteria* (Fig. 5A). No significant enrichment of taxa was observed for the borough the Bronx. Members of *Bacteroidetes* also were significantly elevated in the August samples compared to the others. The August time point showed a higher number (*n* = 10) of significantly different taxon abundances compared to February (*n* = 3) and May (*n* = 2) (Fig. 5B).

### Total 16S rRNA gene abundance in NYC sewage.

**FIG 6:**
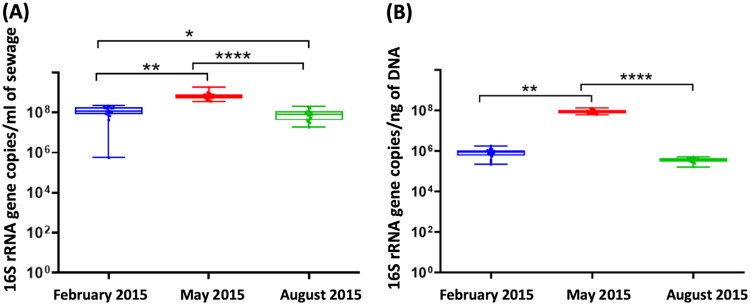
16S rRNA gene copies in NYC sewage samples. (A) Abundances normalized against the volume of sample used. (B) Biomass concentrations of the sewage samples determined by the ratio of the 16S rRNA copies to the total DNA extracted (ng) for each sample. Each box represents a scatter of the mean values of the 17 replicated sewage samples collected at that particular time point. Error bars indicate the standard deviations at each time point. Statistical significance was determined by using a one-factor Friedman’s test, followed by Dunn’s multiple-comparison test (**, *P* < 0.01; ***, *P* < 0.001; ****, *P* < 0.0001).

To assess the variation in total bacterial counts (absolute abundance), quantitative PCR (qPCR) assays were performed to measure the number of 16S rRNA genes present in all 102 samples. The qPCR results showed 10^8^ to 10^9^16S gene copies/ml in all the sewage samples across the three sampling points (Fig. 6). The abundances were highest (Fig. 6A) in the sewage samples from the May time point (mean, 7.1 × 10^8^ copies/sewage ml) compared to February (*P* < 0.01) and August (*P* < 0.0001). The biomass concentration of the samples calculated based on the ratio of the 16S rRNA copies to the DNA yields also was highest in May (Fig. 6B). Within each of the three testing time points, no significant differences were observed between the boroughs or the individual sewage samples.

### Abundance of antibiotic resistance genes.

**FIG 7:**
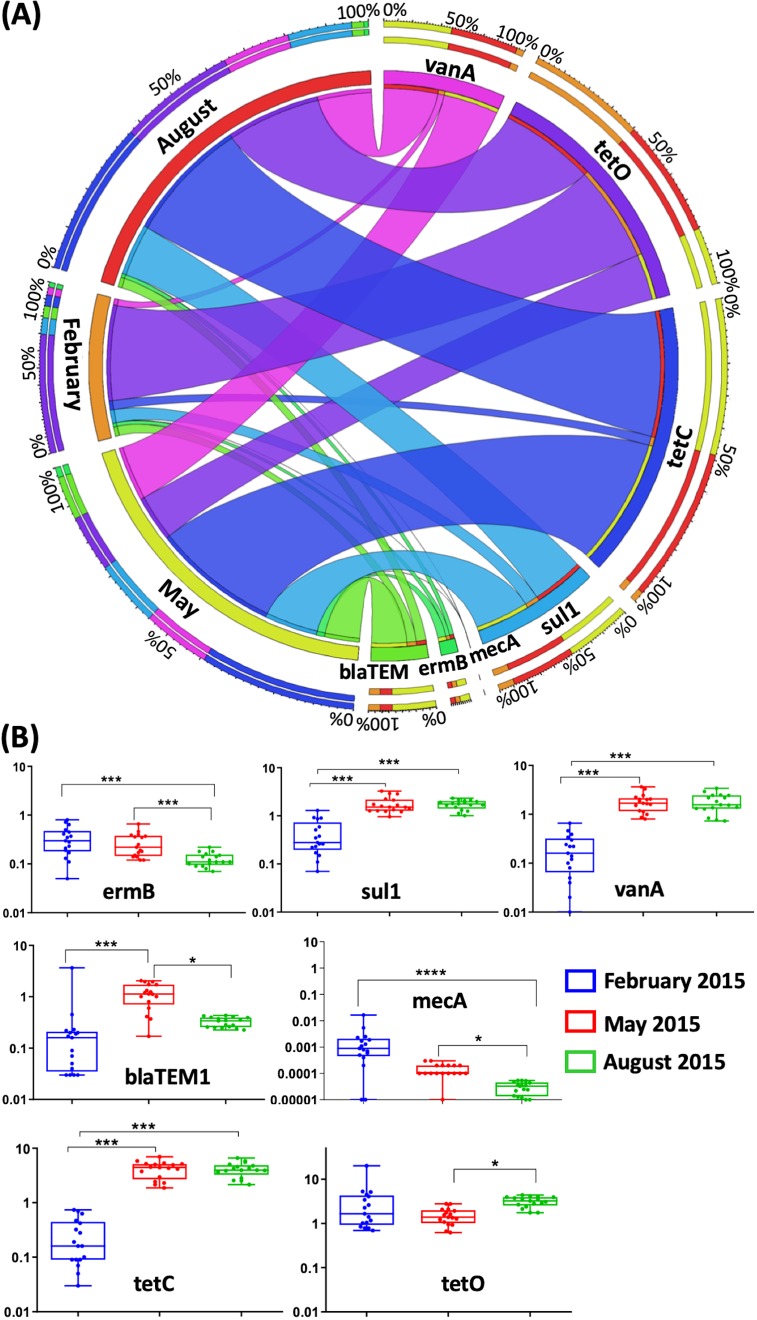
Antibiotic resistance gene representation in proportion to the 16S rRNA gene copies in the NYC sewage samples. Collections were made during February, May, and August 2015. (A) A Circos diagram represents the percent breakdown of the presence of the measured ARG concentrations in each month and vice versa indicated by the connecting ribbons. (B) Ratios were calculated by normalization of antibiotic resistance gene abundance against the 16S rRNA gene abundance for each sample at each time point. Each box represents a scatter of the mean values of the ratios for the 17 replicated sewage samples collected at that time point. The bars indicate the standard deviations at each time point. Statistical significance was determined using a one-factor Friedman’s test, followed by Dunn’s multiple-comparison test (**, *P* < 0.01; ***, *P* < 0.001; ****, *P* < 0.0001).

Our main goal was to assess abundances of indicator antimicrobial resistance genes (ARGs). Using gene-specific qPCR assays, we examined seven ARGs (Table S2) that all confer resistance to commonly used antibiotics, including macrolides (*ermB*), sulfonamides (*sul1*), vancomycin (*vanA*), beta-lactams (*bla*_TEM1_ and *mecA*), and tetracyclines (*tetC* and *tetO*) (8), as well as *mecA*, the most common determinant of MRSA (23). All seven of the resistance genes were present in each of the 102 sewage samples, with variations in concentrations (Fig. 7) and abundances (Fig. S4) across the seasons. For each resistance gene, no significant differences were observed between the NYC boroughs or between the individual sewage samples at each time point; as such, all further analyses were performed by comparing the values of the three seasonal time points. Apart from *mecA*, the other six resistance genes were present at abundances of 10^6^ to 10^8^ copies/sewage ml; *tetC* was detected at the highest mean concentrations (3.4 × 10^7^ copies/sewage ml in May and 3.4 × 10^6^ copies/sewage ml during August (Table 1). During the February sampling, the highest detected mean concentration was for *tetO* (3.72 × 10^6^ copies per ml of sewage). In parallel with the 16S rRNA gene abundances, five antibiotic resistance gene abundances were found to be significantly higher at the May time point compared to February and August. The exceptions were *vanA* and *mecA*, which showed declines in abundances from February to August. The *mecA* gene was detected at the lowest abundance, with a mean of 3.7 × 10^3^ copies/sewage ml in February, decreasing to 2.5 × 10^1^ copies/sewage ml in August. Samples from one WWTP in Queens had low concentrations of most of the genes, particularly in February and August. In general, the resistance gene abundances paralleled those of the 16S rRNA genes in the same samples, with the highest ratios for most in May, followed by August. Tetracycline resistance genes predominated in both months (Fig. 7A), and the ratios were lowest for *mecA*, decreasing from 2 × 10^−5^ (February) to 3 × 10^−7^ (August) (Fig. 7B). Linear regression analysis showed that the 16S rRNA gene abundances significantly correlated (*P* < 0.001) with the abundances of most of the ARGs in the three sampling time points (Table S4).

**TABLE 1:** Summary of the results obtained after quantitative monitoring of the 16S rRNA gene and seven ARGs in the sewage samples obtained from 14 NYC WWTPs over three sampling time points^*a*^

Gene tested	February		May		August	
	Mean gene copies/ml of sewage	Mean % resistance gene/ 16S gene copies	Mean gene copies/ml of sewage	Mean % resistance gene/ 16S gene copies	Mean gene copies/ml of sewage	Mean % resistance gene/ 16S gene copies
**16S**	**2.68E+08**	**NA**	**7.1E+08**	**NA**	**8.13E+07**	**NA**
*mecA*	3.65E+03	0.002	9.1E+02	0.0001	2.51E+01	0.00003
*bla*_TEM-1_	2.00E+05	0.31	7.47E+06	1.17	2.57E+05	0.3248
*ermB*	5.95E+05	0.13	2.27E+06	0.27	1.02E+05	0.13
*tetO*	3.72E+06	3.19	1.06E+07	1.53	2.35E+06	3.148
*tetC*	8.44E+05	0.3	3.04E+07	4.2	3.36E+06	4.15
*vanA*	4.05E+05	0.21	1.22E+07	1.83	1.70E+06	1.81
*sul1*	7.37E+05	0.44	1.2E+07	1.77	1.44E+06	1.72

a NA, not applicable. Boldfacing indicates data for the 16S gene.

10.1128/mSystems.00327-19.4 FIG S4 Abundance of seven ARGs detected in the NYC sewage samples. Collections were made during February, May, and August 2015. Each box represents the scatter of the mean abundance values of the 17 replicated sewage samples collected at that particular time point. The error bars indicate the standard deviations at each time point. Statistical significance was determined using a one-factor Freidman’s test, followed by Dunn’s multiple-comparison test (**, *P* < 0.01; ***, *P* < 0.001; ****, *P* < 0.0001). Download FIG S4, TIF file, 1.1 MB. Copyright © 2019 Joseph et al. 2019 Joseph et al. This content is distributed under the terms of the Creative Commons Attribution 4.0 International license.

10.1128/mSystems.00327-19.6 TABLE S2 Details of the antibiotic resistance genes and primers used in the qPCR studies of the sewage samples. Download Table S2, XLSX file, 0.01 MB. Copyright © 2019 Joseph et al. 2019 Joseph et al. This content is distributed under the terms of the Creative Commons Attribution 4.0 International license.

10.1128/mSystems.00327-19.7 TABLE S3 Complete list of the 73 bacterial families identified from 384 OTUs and their relative abundances based on their sampling locations and season. Download Table S3, XLSX file, 0.03 MB. Copyright © 2019 Joseph et al. 2019 Joseph et al. This content is distributed under the terms of the Creative Commons Attribution 4.0 International license.

10.1128/mSystems.00327-19.8 TABLE S4 Results of linear regression analysis to compare the abundances of the 16S rRNA genes in the population to the abundances of the seven AR genes for the three sampling time points. Analysis was conducted using GraphPad Prism 7 on the log-transformed values of the qPCR data. Download Table S4, XLSX file, 0.01 MB. Copyright © 2019 Joseph et al. 2019 Joseph et al. This content is distributed under the terms of the Creative Commons Attribution 4.0 International license.

## DISCUSSION

One of our priorities was to optimize the DNA yields from sewage samples to ensure optimum coverage of the microbially diverse populations in the samples. Since preliminary assays (24) indicated that for highest yield, genomic DNA extraction from samples should be performed on the day of collection, rather than after storage, we strictly followed that procedure in this study. We used replicate extraction samples in this study to reduce potential sampling and laboratory handling bias during the experimental procedures and, based on the taxonomic summary obtained post-sequencing, the replicates were found to be robust. Since sewage samples comprise domestic, clinical, and environmental waste, we expected the microbial diversity in these samples to be high. For this reason, the samples were split into two sequencing runs (Table S1) to maximize sequencing coverage and depth. The diversity analyses indicated that the sequencing run affected the distribution of the microbial population, causing an artifact involving the OTUs obtained from open reference OTU picking (Fig. S2). Our analysis of the entire study data set using a closed-reference OTU picking strategy avoided false conclusions arising from the batch sequencing run effect, although diminishing the sensitivity of the analysis to detect taxa at low abundances that an open-reference OTU picking strategy would provide. This is relevant to microbiome studies linked with environmental samples, highlighting the utility of combining comparable samples in a single sequencing run, as reported (25).

Beta diversity analysis revealed the bacterial populations to be essentially similar across the boroughs and the three time points, dominated by environmental bacterial taxa compared to human microbiota. This is not surprising since the sewage in these WWTPs is a mix of industrial waste and surface runoff mixed with domestic and clinical wastes. These results are consistent with analysis of sewage from 71 U.S. cities showing that only 15% of the bacterial sequences were of human origin as determined based on their predominance in human gut microbial populations (17). At the family level, the microbial populations observed in this study shared several similarities, e.g., *Bacteroidaceae*, *Lachnospiraceae*, *Porphyromonadaceae*, and *Prevotellaceae*, among others. Both unweighted and weighted UniFrac analyses showed clustering by sampling time, consistent with seasonal variation. New York City is known to have major seasonal differences in temperature (ranging from mean 23.9°F in February 2015 to 79°F in August 2015) (26) that could strongly influence environmental microbial growth dynamics. The 16S rRNA qPCR assays showing significantly higher populations in May 2015 compared to February and August could not be explained by temperature alone, and these other factors, as yet undefined, might play a role. We were able to obtain from the NYC Department of Environmental Protection (DEP) information for other relevant factors such as atmospheric precipitation for the sampling days and the levels of suspended solids and nutrients in the sewage samples, as well as the biological oxygen demand levels (27), but no conclusive correlations were shown between these values and the results observed.

Similar qPCR assays were used to quantitate selected ARGs, based on their observed abundance in prior studies of aquatic and urban environments (28–30). Although all the sewage samples studied tested positive for all assayed ARGs, the May samples had the highest concentrations for five of the seven tested genes, reflecting the higher microbial load in the samples. Since the starting material used for the analysis in this study was quite low at 1 ml, extra precautions, such as the use of replicate samples at each stage, in-plate standards and controls for the qPCR, and accurate optimization of each primer pair, were applied to eliminate the possibility of any artifact formation that could be misleading.

Seasonal variation in antibiotic prescribing in NYC could explain some of the seasonal variation of ARGs, since this May peak in ARG could reflect a lag from the high reported prescription rates in the late winter months, especially in February and March (31, 32). Another factor that could explain the seasonal variation in the levels of antibiotic resistance genes is the influence of the environment and the dilution effect. There is a high level of snowfall in NYC, as well as storm events in the winter months, while the precipitation levels were very low for the spring and summer sampling time points of that year. Since these sampled sewage waters are also inclusive of storm and rainwater runoff, the dilution factors introduced by these environmental influences in winter may also play a role in differing levels of resistance genes in the samples tested. Since the sewer system drained the urban area of NYC, where there is not any farming or animal husbandry, we presume that there was no substantial agricultural contribution to the effluent.

Each of the two tested tetracycline resistance genes (*tetO* and *tetC*) showed high abundances for the three time points sampled. Tetracyclines are widely used in human and veterinary clinical medicine and in food animal husbandry, and resistance genes for tetracycline are also spread across environmental and human samples involving both Gram-positive and -negative pathogens and commensals (33). The high abundances of more than one tetracycline resistance gene in this study are consistent with this ubiquity of the *tet* gene group (16, 34), both as single genes and on mobile genetic elements with multiple resistance cassettes (33).

Although present at significantly lower concentrations compared to the other ARGs, *mecA* was detectable in all samples. In two separate studies of municipal and clinical wastewater in Germany, *mecA* was similarly found at much lower levels than other ARGs tested and was detected only in clinical wastewater samples (35, 36). Since all of the wastewater samples in the present study included sewage released from hospitals, their exact contributions to *mecA* abundances cannot be ascertained. *mecA* has been detected in other WWTP samples (37, 38), but abundances were not quantitated, precluding comparison with our study. A United States-based study of MRSA in wastewater reported 83% positivity of influent sewage samples and 8% positivity of effluent samples (39), reflecting the incomplete ability of wastewater treatment to completely clear this ARG. The presence of *mecA* in the sewage samples may both reflect the presence of community-acquired MRSA infections and potentially contribute to their spread.

Since the sewage samples used in these studies were a mix of different sources in the city, we cannot precisely identify the source of the antibiotic resistance genes detected. Antibiotic resistance in the environment is known to be especially high near urban areas because of the influence of human activities (13). Since the sewage samples in this study derive from both surface runoff and groundwater, and as much of the bacterial population reflects environmental bacteria (Fig. 4), we hypothesize that a substantial proportion of the observed ARGs is of environmental origin. Investigations of ARGs in Danish WWTPs indicated that most had low probability of being associated with human pathogens and represent what has been described as a “sewage resistome” (26).

It is pertinent to note here that qPCR provides measurement of only the resistance genes we targeted to study, making this a minimal estimate of the actual ARG presence. A number of recent sewage-based population studies have employed more sensitive methods, such as shotgun metagenomic sequencing, metagenomic cloning, and gene arrays, to estimate the presence of AMR gene populations in wastewater samples (40–43). Unlike most of these studies, we did not observe any significant geographically distinguishing factors between the samples; any variation observed in our study was primarily based on season (42, 43). Hendriksen et al. conducted a global comparison of AMR presence in untreated sewage samples using a shotgun metagenomic sequencing approach and found correspondence between public health factors such as sanitation and the levels of AMR genes and microbial composition in sewage (41). In another study focusing on sewage samples collected in Hong Kong, nanopore metagenomic sequencing helped reveal mobile genetic elements in bacteria as the predominant source of AMR genes (40). Since qPCR offers the advantage of targeting specific genes, a combination of methods using metagenomic sequencing and qPCR assays of genes of interest could be an important approach for global monitoring of antimicrobial resistance in aquatic environments.

Sewage also can contain residual antibiotic compounds (44) which exert further selective pressure on the environmental bacterial populations. The closed WWTP niche also may select for horizontal gene transfer (45) among the sewage bacteria, especially since many ARGs are present on mobile elements (36) and may be coselected by factors conferring resistance to heavy metals (46), biocides (including triclosan and chlorhexidine) (47), and pharmaceutical compounds. Toxicological data for antibiotic levels in these samples that correspond with sampling times were not available. However, several studies have shown the presence of high levels of pharmaceuticals, including some types of antibiotics, in the aquatic environment of the NYC area that potentially correspond to wastewater discharge and contamination (48, 49).

Although this study focuses only on the microbial and ARG populations in raw, untreated sewage, resistance may also persist after wastewater treatment, with ARGs present in the effluents of sewage treatment plants (44, 50). Since the treated effluents are often released into rivers and other groundwater sources and also being reused in recent times (51), such selective pressures could add to the environmental antibiotic resistance reservoir, highlighting the importance of strict regulations in this regard. Recent studies have reported the unexpected presence of ARGs in locations as isolated as the Arctic, a testament to the ease of widespread dissemination of antibiotic resistance through human activities (52, 53). There are currently no regulations in the United States for monitoring the level of antibiotics or ARGs in aquatic reservoirs or even in drinking water; such rules should be considered as part of broader stewardship efforts.

In conclusion, we report the results from a citywide bacterial diversity and antibiotic resistance study conducted on New York City sewage, showing a stable, generally conserved, bacterial population, and indicator ARGs that were similarly conserved. With increasing global urbanization, citywide studies such as these can help to establish baseline characteristics of the urban microbiome and resistome. These data also can serve as a reference point for further longitudinal studies, comparisons with other locales, and future major extreme weather events.

## MATERIALS AND METHODS

### Sewage collection and DNA extraction.

For this study, raw sewage samples were obtained from DEP WWTPs at three time points: February, May, and August 2015. For each time point, 17 samples were collected from the 14 NYC WWTPs, representing the five NYC boroughs (Fig. 1); samples from three of the WWTPs were split to represent smaller drainage areas within these plants, resulting in a total of 17 samples (27). Two of these samples also were from drainage areas shared across boroughs and thus have been indicated as “Bronx_Manhattan” and “Brooklyn_Queens” in the data (see Table S1 in the supplemental material). Each sample was a composite of sewage obtained over a 24-h period from each plant. Additional details of the raw sewage collected from the WWTPs are described in Maritz et al. (27).

Samples were provided in 200-ml containers, from which 1-ml volumes were used for DNA extraction. During earlier protocol optimizations, the majority of the samples were found to be densely particulate, which clogged the filter membranes during passage of small sewage volumes. After yield comparisons with centrifugation processes (24), the protocol was optimized to use 1 ml of sewage sample. Each sample was then subjected to rigorous bead beating for ∼20 min using 0.7-mm garnet beads, followed by nucleic acid extraction using Powersoil DNA extraction kits (Mobio, Carlsbad, CA), according to the manufacturer’s instructions. Duplicate extractions were performed simultaneously, and all processing and extraction procedures were completed on the day of sampling for standardization and to ensure maximum DNA yields. Nucleic acid quantitation was carried out with the Qubit 2.0 (Thermo Fisher Scientific, Waltham, MA), and the samples were stored at –20°C.

### 16S rRNA library preparation and sequencing.

For each DNA sample, the V4 region of the 16S rRNA gene was amplified using the primer set 515F/806R (54) with 12-bp barcodes in triplicate reactions. These standard bacterial primer sets have been used in other large-scale community studies (55). Each 25-μl PCR reaction mixture contained 10 μl of 5 Prime Hot Master mix (5-PRIME), 1 μl each of the forward and reverse primers (5 μM), 10 μl of molecular-biology-grade distilled water, and 3 μl of each genomic DNA template. Reaction negative controls, as well as kit extraction controls, were amplified simultaneously. The PCR conditions were as follows: 94°C for 3 min; 35 cycles of 94°C for 45 s, 50°C for 1 min, and 72°C for 1.5 min; followed by a final extension step for 10 min at 72°C. The amplified triplicate reactions were pooled and quantified using a Quant-iT PicoGreen dsDNA assay kit (Invitrogen, Waltham, MA), and then equimolar concentrations of each amplified sample were combined into a pool that was purified by using a QIAquick PCR purification kit (Qiagen, MD). The pooled amplicons were sequenced on an Illumina MiSeq 2500 instrument using 2 × 150 paired-end sequencing at the NYU Genome Technology Center. The samples were split into two batches to obtain a higher sequencing depth to maximize capturing diversity. The distribution of the samples across the two sequencing runs and PCR is indicated in Table S1. PCR controls were included in each run.

### Bioinformatics analysis.

Bioinformatic processing was performed using QIIME v1.9.1 (56) with parameters as described previously (57). Briefly, mate-pair reads were joined using fastq-join (58) of the fastq_join.py command, and reads with at least a 40-nucleotide (nt) overlap between mate-pairs were retained for further quality filtering. The remaining sequences were truncated at any site containing >3 consecutive bases receiving a quality score of <20, and any read containing one or more ambiguous base call was discarded, as were reads <190 nt in length. Samples were then demultiplexed, and OTUs were picked first using the open reference and then the closed-reference OTU picking methods in QIIME. Reads were clustered into 97% identity OTUs using UCLUST (v1.2.22), followed by taxonomy assignment using the RDP classifier (v2.2) against the Greengenes database version 13.8 (59) with a 0.5 confidence (60). All singleton OTUs were removed and chimeric sequences were then removed using ChimeraSlayer (61). The resulting OTU table was filtered to remove OTUs with a relative abundance of <0.01% across all samples to eliminate minor taxa (57) and was used in all subsequent analyses.

Diversity analyses were performed using the QIIME script core_diversity_analyses.py. Alpha diversity was calculated within QIIME using the Shannon-index, PD (phylogenetic diversity) tree, and observed OTUs. Rarefaction plots were subsampled to the lowest sample sequencing depth of 58,892 sequences/sample, and significance was determined using a two-sample nonparametric *t* test with 999 Monte Carlo permutations and corrected for multiple comparisons using Bonferroni correction. Beta diversity was assessed using unweighted and weighted UniFrac distances, and ordinations were plotted using PCoA in the Phyloseq and ggplot2 packages in R (62, 63). Significance was determined using the nonparametric permutational MANOVA (PerMANOVA) Adonis test with 999 permutations using the Vegan package (v2.5-2) in R (64). Linear discriminant analysis effect size (LEfSe) analysis (65) was performed at the family level on the filtered OTU table using an alpha value of <0.05 and a Linear Discriminant Analysis (LDA) score greater than 4.0. Taxonomic abundance plots were generated using the R packages, Phyloseq, and ggplot2. OTUs were summarized at the family level with a minimum relative abundance of 3% in each sample.

### Preparation of standards for qPCR.

The antibiotic resistance genes for qPCR were selected based on resistance to some of the more commonly used antibiotics and their abundances observed in prior studies of aquatic and urban environments (28–30). The reference standards for the qPCR assays were constructed by cloning a large construct of each resistance gene into a suitable vector backbone, followed by confirmation of sequence and orientation. The standards for the 16S rRNA and *mecA* genes were cloned on a pGEM-T Easy (Promega) vector backbone and obtained from Zhan Gao (66). Commercially purchased pBR322 (NEB) was used as a reference standard for the *bla*_TEM_ and *tetC* genes. The standards for the other resistance genes were constructed for this study using the pGEM-T Easy vector for cloning the reference genes. The full-length genes for *bla*_SHV_ and *sul1* were isolated from one of the sewage samples used in this study. The *ermB* gene was amplified from an NYU in-house strain of S. aureus (B. Shopsin, unpublished data). The *vanA* gene was amplified from E. faecium DSMZ 17050, while the *tetO* gene was amplified from the fecal DNA from mice treated with tetracycline (67). Each PCR contained 5 μl of the 10× buffer, 1 μl of a deoxynucleoside triphosphate mix, 5 μl each of the forward and reverse primers, 0.25 μl of *Taq* polymerase, and 1 μl (∼10 ng) of genomic DNA sample adjusted to a final volume of 50 μl using molecular-biology-grade dH_2_O. All the primers for the full-length amplification of these genes (Table S2) were synthesized by Integrated DNA Technologies (Coralville, IA). The PCR conditions were as follows: (i) initial denaturation at 95°C for 2 min; (ii) 35 cycles of 95°C for 30 s, an annealing temperature as listed in Table S2 for 45 s, and 72°C for 1 min; and (iii) final elongation at 72°C for 7 min. The amplified PCR products were confirmed using 2% agarose gel electrophoresis and then purified using the QIAquick PCR purification kit. The purified products were then used to construct standards by cloning using the pGEM-T Easy vector system (Promega, WI).

### Quantitation of resistance genes in the sewage samples.

The quantitation of the 16S rRNA genes and antibiotic resistance genes in the sewage samples were performed by PCR amplification on a LightCycler 480 Instrument II (Roche Diagnostics, IN). qPCR reactions of 20-μl volumes each were set up using the LightCycler 480 SYBR green I Master hot-start reaction mix. The primers used and their annealing temperatures are listed in Table S2 and were synthesized by Integrated DNA Technologies. The amplification conditions for the qPCR program were as follows: 40 cycles of 95°C for 10 s, respective annealing temperatures for 20 s, and the fluorescence acquisition step at 72°C for 20 s. Tenfold dilutions of the standards (with copy numbers ranging from 10^8^ to 10^0^ ) for each gene were used to first plot the standard curve and then to optimize the primers to ensure that acceptable efficiencies of 90 to 110% were maintained for each set of primers. Baseline and melting curve analyses were performed using the LightCycler 480 software (Roche). Each reaction was carried out in duplicate wells, and each run was performed with replicate samples and standards on the same plate to maintain uniformity. Since some of the data sets did not exhibit normal distribution, a nonparametric one-factor Friedman’s test was used to estimate any statistically significant differences within the means of the groups in the population for each gene tested. This was followed by the Dunn’s multiple-comparison tests to compare the *P* values and statistical significances between the three time points in each gene set. Linear regression analysis was performed to calculate the relationship between the abundances of the 16S rRNA genes and AR genes using log-transformed values. All statistical analyses were performed using GraphPad Prism 7. Visualization of the ARG qPCR data was performed using GraphPad Prism 7 and Circos Table Viewer v0.63-9 (52, 68).

### Data availability.

All the MiSeq sequencing output fastq files and metadata are publicly available via the NCBI Sequence Read Archive (SRA) under accession no. PRJNA542507.
