# Supplementary figures and images for: Longitudinal Comparison of Bacterial Diversity and Antibiotic Resistance Genes in New York City Sewage
Source: mSystems. 2019 Aug 6;4(4):e00327-19. doi: 10.1128/mSystems.00327-19 (PMC6687945; doi:10.1128/mSystems.00327-19)

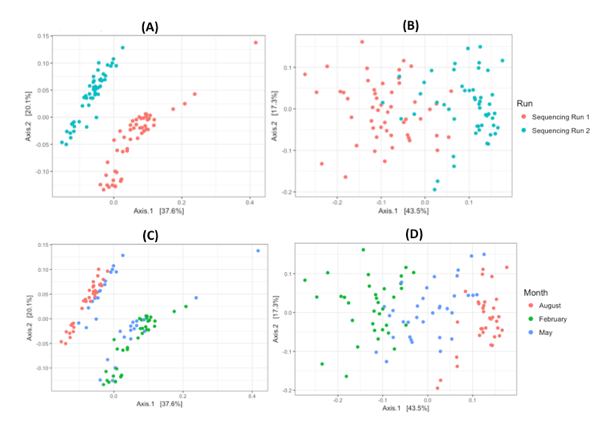

Supplement: FIG S1 [file mSystems.00327-19-sf001.tif]

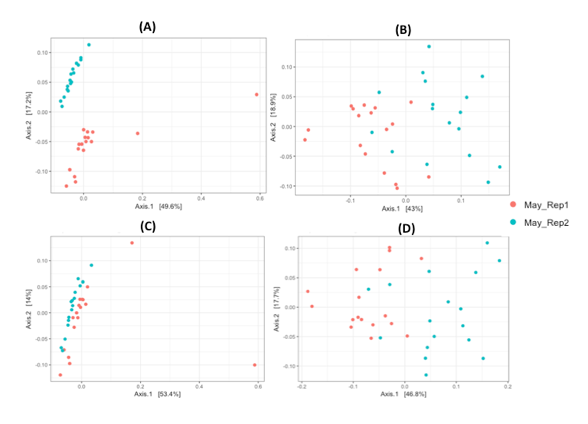

Supplement: FIG S2 [file mSystems.00327-19-sf002.tif]

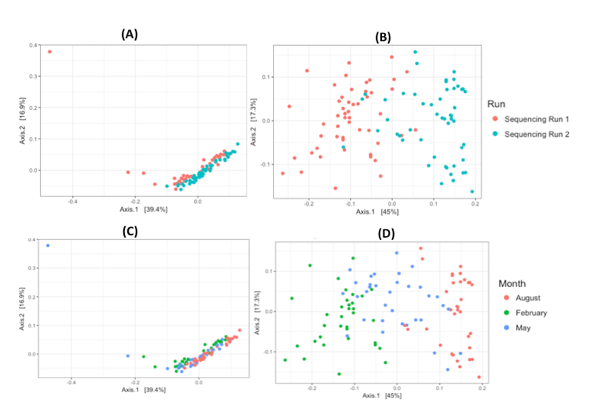

Supplement: FIG S3 [file mSystems.00327-19-sf003.tif]

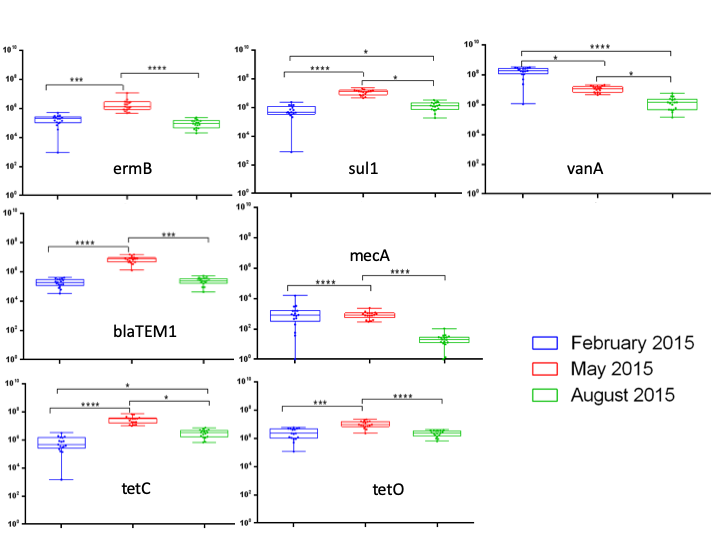

Supplement: FIG S4 [file mSystems.00327-19-sf004.tif]
